# Robust Inferential Techniques Applied to the Analysis of the Tropospheric Ozone Concentration in an Urban Area

**DOI:** 10.3390/s21010277

**Published:** 2021-01-03

**Authors:** Wilmar Hernandez, Alfredo Mendez, Vicente González-Posadas, José Luis Jiménez-Martín, Iván Menes Camejo

**Affiliations:** 1 Facultad de Ingeniería y Ciencias Aplicadas, Universidad de Las Américas, Quito EC-170125, Ecuador; 2 Departamento de Matematica Aplicada a las Tecnologías de la Informacion y las Comunicaciones, ETS de Ingeniería y Sistemas de Telecomunicacion, Universidad Politecnica de Madrid, 28031 Madrid, Spain; alfredo.mendez@upm.es; 3 Departamento de Teoría de la Señal y Comunicaciones, ETS de Ingenieria y Sistemas de Telecomunicacion, Universidad Politecnica de Madrid, 28031 Madrid, Spain; vicente.gonzalez@upm.es (V.G.-P.); joseluis.jimenez@upm.es (J.L.J.-M.); 4 Facultad de Informatica y Electronica, Escuela Superior Politecnica de Chimborazo, Riobamba EC-060155, Ecuador; imenes@espoch.edu.ec

**Keywords:** robust analysis, central tendency and scale estimates, robust and nonrobust confidence intervals, categorization of ozone measurements

## Abstract

This paper analyzes 12 years of tropospheric ozone (O_3_) concentration measurements using robust techniques. The measurements were taken at an air quality monitoring station called Belisario, which is in Quito, Ecuador; the data collection time period was 1 January 2008 to 31 December 2019, and the measurements were carried out using photometric O_3_ analyzers. Here, the measurement results were used to build variables that represented hours, days, months, and years, and were then classified and categorized. The index of air quality (IAQ) of the city was used to make the classifications, and robust and nonrobust confidence intervals were used to make the categorizations. Furthermore, robust analysis methods were compared with classical methods, nonparametric methods, and bootstrap-based methods. The results showed that the analysis using robust methods is better than the analysis using nonrobust methods, which are not immune to the influence of extreme observations. Using all of the aforementioned methods, confidence intervals were used to both establish and quantify differences between categories of the groups of variables under study. In addition, the central tendency and variability of the O_3_ concentration at Belisario station were exhaustively analyzed, concluding that said concentration was stable for years, highly variable for months and hours, and slightly changing between the days of the week. Additionally, according to the criteria established by the IAQ, it was shown that in Quito, the O_3_ concentration levels during the study period were not harmful to human health.

## 1. Introduction

Ozone is a substance that, based on its composition, is classified as a simple molecular substance made up of three oxygen atoms joined by covalent bonds, one single and the other double; its molecular formula is O_3_ [1,2].

According to [3], the ozone molecule is angular, with an angle of 117° and a structure in resonance between two possible electronic configurations. It is diamagnetic, indicating the absence of unpaired electrons [3].

Ozone (O_3_), whose name is trioxygen according to [4,5], is one of the allotropes of oxygen, the most familiar of which is molecular oxygen (O_2_), also known as dioxygen. Its CAS Registry Number (Chemical Abstracts Service registration number) [6] is 100-15-6.

### 1.1. Stratospheric Ozone

Ozone is concentrated in the stratosphere between 12 and 25 km high, forming the ozone layer, and reaching its maximum concentration at a height between 20 and 25 km. Stratospheric ozone is formed by the action of ultraviolet radiation, which dissociates the dioxygen molecules into two highly reactive atoms, as in Equations (1) and (2), which react with the molecular:

(1) $$O_{2}+h\nu (\lambda <240\;\mathrm{nm})\to 2O$$

(2) $$O_{2}+O+M\to O_{3}+M,$$

where O_2_ is oxygen, λ stands for wavelength, the photon’s energy is E=hν, the Planck constant is h, the photon’s frequency is ν, and O is an oxygen atom. In addition, (2) occurs with heat release and requires the presence of a third body, M, such as N_2_ (dinitrogen) or O_2_, which removes the energy from the reaction and stabilizes the ozone molecule.

Ultraviolet radiation with a wavelength greater than 290 nm causes the O_3_ molecule to dissociate as shown in Equations (3) and (4):

(3) $$O_{3}+h\nu \left(\lambda >290\;\mathrm{nm}\right)\to O_{2}+O$$

(4) $$O+O_{3}\to 2O_{2}$$

This set of processes is called the Chapman cycle [7].

A dynamic equilibrium is formed in which ozone is formed and destroyed, consuming most of the wavelength radiation less than 290 nm. Thus, ozone acts as a filter that does not let harmful radiation pass through to the Earth’s surface [8].

In accordance with [7], due to stratospheric winds, ozone is transported from tropical to polar regions. Thus, the closer human beings live to the equator, the less ozone will protect them from ultraviolet light.

The ozone balance in the stratosphere is affected by the presence of pollutants known as ozone-depleting substances, such as, among others, chlorofluorocarbons (CFCs), which rise to the upper atmosphere and catalyze the destruction of ozone faster than it is generated, thus producing a hole in the ozone layer [9].

### 1.2. Tropospheric Ozone

Tropospheric ozone, also called environmental ozone, is a colorless gas that is mainly created through photochemical reactions of nitrogen oxides (NOx) and volatile organic compounds (VOCs) [10]. Additionally, soil and plants emit NOx and VOCs (biogenic sources) [11].

O_3_ is formed through reactions caused by sunlight. The phenomenon by which precursors are emitted and accumulated through reactions necessary for the formation of ozone to occur is called photochemical smog [12]. Smog episodes are the main production process for tropospheric ozone [13]. It is the most important oxidant present in the lower levels of the atmosphere, making it a potentially dangerous compound due to its ability to react with most compounds, degrading them. In addition, this affects both materials and living things that are affected by its action, externally but also internally, in the gas exchange that takes place when breathing, which is the main way humans are affected by ozone pollution. The response also varies greatly between individuals for genetic reasons (antioxidant capacity of cells), age (children and the elderly are the most sensitive groups), and the presence of respiratory conditions (allergies and asthma) [14].

When the ozone concentration is high, it is not advisable to do physical exercise, especially in the central hours of the day, since it increases the breathing rate and increases the entry of ozone into the lungs [11,14]. In regards to vegetation, a high level of ozone damages the leaves, reduces plant growth, and leads to a lower crop yield [11,14]. In the vicinity of the sources (cities, roads, and industries), fresh emissions can react with ozone and locally reduce its concentration; nitric oxide emitted into the air locally by automobiles removes ozone carried from other parts [3]:

(5) $$\mathrm{NO}+O_{3}\to {\mathrm{NO}}_{2}+O_{2}$$

where NO is nitric oxide and NO_2_ is nitrogen dioxide. However, at a certain distance, the photochemical formation of O_3_ is reactivated, which is why the concentrations of O_3_ are normally low in industrial centers and urban areas. On the other hand, in rural areas and in the outskirts of urban areas, the concentration is higher [11].

The photochemical formation of ozone does not occur during the night, because there is no sunlight [11]. In addition, during the day, the maximums of O_3_ concentrations occur from noon, when the radiation is highest; regarding the time of year, the maximums occur during the spring and summer months.

The World Health Organization [15] has established that when the ozone concentration in the air that is breathed is higher than $240\;\mu g/m^{3}$ and is maintained for more than 8 h, there is a probability of significant health effects [16].

To synthesize ozone, a machine called an ozone generator or ozonizer is used, in which a high electrical voltage known as the corona effect is generated, which produces ozone from O_2_ [17]. The ozone generation has application in the elimination of bad odors and disinfection of the air, in the treatment and purification of water, and in ozone therapy.

What has been mentioned in the previous paragraphs provides a justification for carrying out a robust analysis [18,19,20] of the information given by ozone measuring systems, so that we can be able to describe with high precision the behavior of ozone concentration in cities, even when there are a large number of extreme observations and the data under study follow heavy-tailed distributions [19,21]. This last aspect has become a bottleneck because many times the outliers represent the response of the physical system to certain types of inputs. Therefore, these values are carriers of useful information and should not be eliminated, but must be treated appropriately in order to understand what they are telling us about the system from which they come. It is for this reason that the robust data analysis gains strength and shows a highly precise way to explain the behaviors of air pollution variables, even when they come from heavy-tailed distributions with a large number of outliers.

The aforementioned, applied to the analysis of ozone concentration in urban areas, constitutes one of the contributions of the research presented in this paper.

The robust analysis of the concentration of ozone at Belisario air quality monitoring station, which is in Quito, Ecuador, is the foremost objective of this research. This analysis is carried out from 1 January 2008 to 31 December 2019 [22,23].

Quito was chosen because of the effect of this city’s vehicular traffic, poor fuel quality, and traffic jams on air pollution levels. Furthermore, there are other factors in the city that could increase air pollution levels, such as industrial zones and population growth [23].

The design of a sensor-based system aimed at measuring and georeferencing atmospheric variables, including ozone, is presented in [24]. In addition, a low-cost air quality monitoring station was presented in [25], where the authors proposed a calibration procedure based on artificial intelligence techniques. Furthermore, another air quality monitoring system was presented in [26], which employed a Zigbee network to improve the network layout. Additionally, a long short-term memory (LSTM) network was used in [26] to predict the urban air quality pollution period. Moreover, O_3_ and seven other indicators were used in [26] to qualitatively validate the experiential knowledge of the authors; to allow cells to have long-term memory, the hidden layer of recurrent neural network cells was replaced by LSTM cells.

A meta-analysis of ground-level ozone pollution was carried out in [27]. This analysis was performed to find the similarities and differences between the risk of asthma aggravation and ground-level ozone exposure measurements. In [27], three different time periods (i.e., 1-h daily maximum, 8-h daily maximum, and 24-h average concentrations) were used. In addition, the meta-analysis performed in [27] was carried out using pooled relative risks, 95% confidence intervals, and random-effects models [28]. Additionally, in order to achieve robustness in the results, subgroup and sensitivity analyses were conducted in [28]. Moreover, the Cochrane Q statistic and I^2^ estimation [29] were used to measure heterogeneity and inconsistency in the meta-analysis. Furthermore, in order to test whether there was publication bias, the authors made use of a funnel plot and Egger’s test [30].

In [31], a study was carried out in 48 cities of China from 2013 to 2017. In that study, time-series analyses were conducted and the authors used a generalized model combined with a random effect model to estimate ozone levels. Another time-series study was performed in [32] in 184 cities of China from 2014 to 2017. Additionally, in [32], the relationship between patients due to pneumonia and ozone concentration was found by using a generalized additive model, and the authors provided robust proof of the existence of a relationship. The robustness of the relation was tested by fitting two-pollutant models.

A 10-year study on the relationship between particulate matter and ozone exposure (PM and OE), and a depression and anxiety diagnosis (DAD) in Saxony, Germany, was conducted in [33]. In that research, the data used for the analysis corresponded to the information collected from 2005 to 2014. In [33], the analysis was performed by using generalized estimating equations [34], and the robust metric was the number of days for which the maximum value of the 8-h average ozone concentration was greater than $120\;\mu g/m^{3}$. Additionally, two-pollutant models were built and a sensitivity analysis was carried out, aimed at studying the relationship between PM and OE and DAD.

The reasons why there was a high ozone concentration in Chengdu, China in July 2017 were studied in [35]. In order to perform such a study, both measurements and air quality simulations were used. Additionally, in order to identify the VOC sources and perform the quantification of these sources, positive matrix factorization [36,37,38] was used. Moreover, in [35], the impact of physical and chemical processes on ozone concentrations was analyzed by the integrated process rate method [39,40,41].

In various situations in which it is intended to study the behavior of air pollution variables, researchers face the problem that these variables either do not follow a Gaussian distribution or do not follow any known parametric distribution. Therefore, classical statistical inference methods cannot be used for data analysis and nonparametric statistical inference must be used. For example, both Mann–Whitney U and Kruskal–Wallis tests [42,43] were applied in [44] to analyze vehicle emissions. In [44], 1000 vehicles were tested in order to find significant differences between the mean emissions of air pollutants. Other instances of authors using robust techniques to analyze the concentration of air pollutants are presented in [45,46,47,48,49,50,51,52,53,54].

In this research, the measurement results of 12 years of tropospheric ozone concentration were analyzed using robust techniques. The urban area chosen for the study was Belisario station [22], and robust statistics [18,19,20] were used in this research to determine the robust central tendency and scale estimations of tropospheric ozone and find parametric, nonparametric, and robust confidence intervals that explain the O_3_ concentration [18,19,20,42,43]. The O_3_ concentration measurements that were analyzed in this paper were taken from 1 January 2008 to 31 December 2019.

The analysis presented here allowed us to judge the central tendency of the data based on the variability. Here, the data were grouped and classified, and similarities and differences were determined. Moreover, confidence intervals were used to do the aforementioned classification and several methodologies were used to analyze the data: classic, nonparametric, bootstrap, and robust methodologies.

In [23], another analysis of air pollution variables in Quito is presented. However, the analysis performed in [23] is not robust and, what is more, it is only based on the mean and maximum values. For this reason, the study performed in this research can be considered essential to comprehensively understand, in a formal and rigorous manner, the behavior of tropospheric ozone at Belisario monitoring station.

In this paper, in order to perform robust data analyses, each year under study was considered as a random variable. Additionally, it was shown that the distribution of these variables was heavy-tailed [19,21]. The concentration of other air pollution variables in Quito was robustly analyzed in [47,48,49,50,51]. Furthermore, in [55,56] some robust estimators were also used. Additional research in which statistical tools have been used to analyze the O_3_ concentration are as follows.

A pollution weather prediction system was proposed in [57] and used to measure O_3_ among other pollutants. In [57], in order to carry out predictions, linear regression and artificial neural networks were used.

An air quality monitoring network aimed at analyzing the changeable nature of ozone across several communities of California, USA, was shown in [58], where the mean absolute error was used to analyze O_3_ concentrations and the accuracy of measurement nodes and their correlation to reference instrumentation was indicated by using least squares regression. Moreover, summary statistics based on the mean, standard deviation, minimum, maximum, mean bias deviation, mean absolute deviations, and ordinary least squares statistics were used in [58] to present the data in a meaningful way.

In order to produce well-calibrated data, both multiple linear regression and nonlinear techniques were used in [59]. Additionally, a recalibration was done to mitigate the bias presented by the sensors and improve the variance.

A low-cost air quality monitoring system was presented in [60]. This system was aimed at monitoring O_3_, among other pollutants, and a comparative analysis between a neuro-fuzzy system and a multilayer feed-forward perceptron was performed.

Finally, a statistical analysis of ensembles of O_3_ profiles at the P. N. Lebedev Physical Institute, Moscow, Russia from 1996 to 2017 was carried out in [61]. This analysis was based on radiometric ozone monitoring and several statistical parameters were calculated: mean, variance, root-mean-square error, probability density function, probability distribution function, covariance function, correlation function, and frequency spectra.

The contribution of the present research with respect to the studies mentioned above is that, in order to optimize the sampling process to reduce power consumption in cases where researchers use portable devices powered by a battery, variables have been defined that represent the hours of the day in which the ozone concentration is the highest. This was done for both hours grouped by months and hours grouped by days of the week, separating working days from weekends, and it was shown that all the variables considered were different. For example, what happens in a particular month has nothing to do with what happens in other months. Therefore, it is difficult to make predictions since the distributions of the variables are different. Specifically, the variables do not come from the same statistical populations. Therefore, it is shown here that using robust methods is more effective than using nonrobust ones.

The objectives of this paper were as follows:

1. Compare the values of tropospheric ozone measurements based on four sets whose elements are variables that represent the following: (a) the 12 years under study, (b) the months: January to December, (c) the days: Monday to Sunday, and (d) the hours in pairs: from 0:00–1:00 to 22:00–23:00.
2. Analyze the behavior of the abovementioned variables in comparison with the different categories of air pollution established by the IAQ of Quito [23].
3. Estimate the data’s central tendency and variability, and quantify differences using robust and nonrobust confidence intervals.

In this paper, it is shown that for the data under study, the trend of tropospheric ozone concentration at Belisario station has been towards stability for years, very variable by months and during the day, and moderately changing between the days of the week.

Some general comments on ozone sensors are made in Section 2. Section 3 describes the data and presents summary statistics on the collected data. Furthermore, nonparametric statistical inference is used in Section 4 to classify the data. Moreover, Section 5 is a robust data analysis and classification, and Section 6 is the discussion. Finally, Section 7 presents the conclusions of the paper.

## 2. Some General Comments on Ozone Sensors: Characteristics and Considerations of Signal Conditioning and Processing

In terms of low-cost O_3_ measurements, the electrochemical (EC) sensor technology and the heated metal oxide sensor (HMOS) technology are at the vanguard [62]. Furthermore, the ultraviolet absorption principle-based analyzer is a well-known method for conducting O_3_ measurements. However, the cost of O_3_ analyzers is prohibitive [62].

In this research, O_3_ measurements were carried out using THERMO 49C/49i photometric O_3_ analyzers [63,64], which are used as measurement standards in several countries (e.g., these instruments are designated by the United States Environmental Protection Agency as a reference [65]). The principle of operation of the models 49C/49i is based on the fact that, at a wavelength of 254 nm, O_3_ molecules absorb ultraviolet light.

The Beer–Lambert Law explains this type of absorption [63,64]. In short, a sample is introduced into the equipment and divided into two gas streams. After passing through an O_3_ scrubber, one gas is used as the reference and sent to a solenoid valve. The other gas that is not sent to the O_3_ scrubber is used as the sample gas and sent to another solenoid valve. Next, the reference gas and sample gas are deposited into two different cells and detectors are used to measure the ultraviolet light intensity of each cell. During the process of switching the sample gas, which is contained in one cell, and the reference gas, which is contained in the other cell, ultraviolet light intensity measurements are not performed until the two cells are flushed. Finally, both the O_3_ concentration of each cell and the average concentration are calculated [63,64].

With regard to low-cost O_3_ measurements, the principle of operation of EC and HMOS sensors is explained in [62]. Some of the strengths of EC sensors are as follows [62]: low power consumption, good repeatability and accuracy, and linear response, among others. On the other hand, some weaknesses of these sensors are that their readings can be affected by humidity and changes in temperature, they have a limited life, and their aging process is fast [62].

Furthermore, some of the strengths of HMOS sensors are as follows [62]: long life, very responsive to O_3_ levels below 0.1 ppm, and excellent repeatability and accuracy. On the other hand, some weaknesses of these sensors are that their response time is slower than that of EC sensors, they require higher power consumption than EC sensors, and the linearity is reduced above 1 ppm [62].

In addition, when health and safety monitoring below 0.1 ppm is required, HMOS sensors can be used. However, when this monitoring is needed above 0.1 ppm, then EC sensors can be used. Moreover, EC sensors can be used to detect O_3_ leaks, in controlling alarms and in O_3_ generators (above 0.1 ppm). Additionally, HMOS sensors can be used in O_3_ control applications below 0.1 ppm, when portable monitoring below 0.1 ppm is needed, and to measure ambient O_3_ concentrations [62].

For the purpose of this research, in order to determine real-time O_3_, a cumulative gas sensor is shown in [66]. Such a sensor used visible spectroscopy, which was based on cheap material, and was aimed at carrying out O_3_ measurements in workplaces using a portable device.

At 600 nm, the abovementioned sensor showed high sensitivity at low O_3_ concentrations, and good reproducibility and stability. In addition, a review of deep ultraviolet absorption applied to gas sensing is presented in [67], and a discussion of applications of ultraviolet absorption spectrometry for O_3_ and other air pollutants is given in [67].

The performance of EC sensors is studied in [68], and it is shown that devices based on these sensors can be built and used at both lower cost and power consumption than conventional O_3_ monitoring devices.

A review of gas and dissolved ozone sensors are presented in [69]. The different principles of operation of the sensors mentioned in [69] are based on the following: amperometric measurement methods, impedimetric measurement methods, and optical measurement methods. Moreover, this review was done in order to establish the requirements that these sensors must meet in order to be used in medical applications. Ozone has also found a large number of applications in the search for solutions to SARS-CoV-2/COVID-19 [70,71].

Furthermore, another review of absorption spectroscopy-based O_3_ sensors is given in [72]. In that paper, sensor applications, performance, limitations, and costs are discussed. Additionally, requirements such as accuracy, dynamic range, response, sensitivity, and cross-sensitivity are discussed for several specific applications.

However, it is not enough to design a sensor that measures the value of a certain physical quantity; this measurement must also be as robust as possible against disturbances and unwanted information. In this sense, several authors [66,67] are also concerned about the robustness that the sensors could have and are aware of the limitations of the sensor as an isolated element. Therefore, to achieve a good design of measurement equipment, it is necessary to put the sensor inside a signal conditioning and processing system that is capable of dealing with everything that represents noise, disturbance, and unwanted information [73]. Additionally, the stage of signal conditioning and processing must transform the sensor signal in such a way that it meets the design requirements of the measuring system.

It must be added that sometimes the systems that are designed to optimally solve the problem of conditioning and processing the signal from the sensors are not causal systems [74]. Therefore, it is necessary to resort to advanced design tools to make these systems realizable. However, the more one tries to design an optimal and robust system, the more complex and expensive the final design becomes. Therefore, it is recommended to establish a tradeoff between the desired performance of the signal conditioning and processing system and what can physically be achieved satisfactorily. Once this tradeoff is established, engineers and researchers can later use other analysis tools that allow them to robustly extract the relevant information from the signal under study. In general, in this last stage, the analysis of the information is carried out after the measuring system delivers the results to the user. Then, with the measurement results at hand, researchers and engineers focus on studying the characteristics of the data, the modeling, and the possibility of predicting the behavior of the physical quantity under study. In some cases, the aforementioned is done in order to design a robust feedback control system [75].

**Figure 1:**
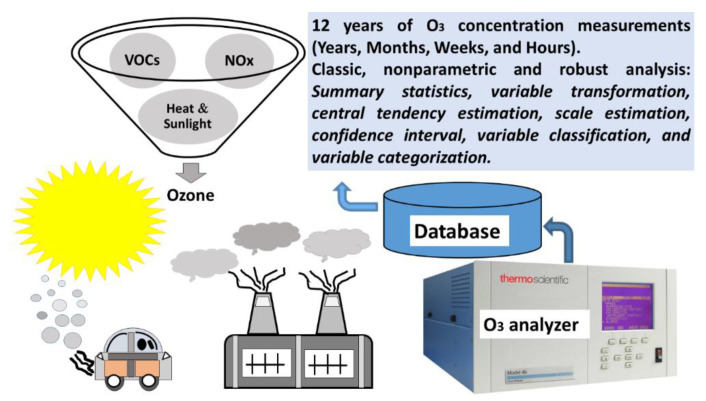
General system diagram.

In this research, THERMO 49C/49i photometric O_3_ analyzers were used to carry out the ozone measurements and, in order to extract the most important information from the measurement results, robust analysis methods were proposed. The proposed methods allowed us to classify and categorize variables that represented sets of ozone measurements, which were taken over hours, days, months, and years. Figure 1 shows the system diagram.

The type of analysis of ozone measurements developed in this paper could serve as a starting point for design procedures that allow researchers to examine in detail the behavior of ozone in different parts of a city.

## 3. Data Description and Summary Statistics of Tropospheric Ozone Measurements at Belisario Station

Here, the ozone measurements were taken using THERMO 49C/49i photometric O_3_ analyzers. The sampling rate was 1 h [23], which would imply that 105,193 data points were obtained. However, some observations could not be recorded because the data were not stored. The database was improved to take this circumstance into account, so it was possible to work with more than 96% of the data. In this way, it was guaranteed that more than 75% of the data were used for the analysis [76].

Due to the fact that the geographical and meteorological characteristics of Quito are adequate to have high insolation, the highest tropospheric ozone concentrations occur in August and September [23]. In addition, the months of the year that are either close to the equinox or correspond to the equinox are the abovementioned months, which are months in which the skies are clear. On the other hand, the months with the lowest tropospheric ozone concentrations in Quito are May and June, which are the months in which there are the most days with cloudy skies and rain [23].

In order to find possible relationships between the data, the following variables were considered:

- $X_{k}$, k=1,⋯,12, where $X_{1}=2008,\;X_{2}=2009,\;\ldots ,\;X_{12}=2019$.
- $Y_{k}$, k=1,⋯,12, where $Y_{1}=$ January, $Y_{2}=$ February, …, $Y_{12}=$ December.
- $Z_{k}$, k=1,⋯,7, where $Z_{1}=$ Monday, $Z_{2}=$ Tuesday, …, $Z_{7}=$ Sunday.
- $W_{k}$, k=1,⋯,12, where $W_{1}=0:00–1:00,\;W_{2}=2:00–3:00,\;\ldots ,\;W_{12}=22:00–23:00$.

**Figure 2:**
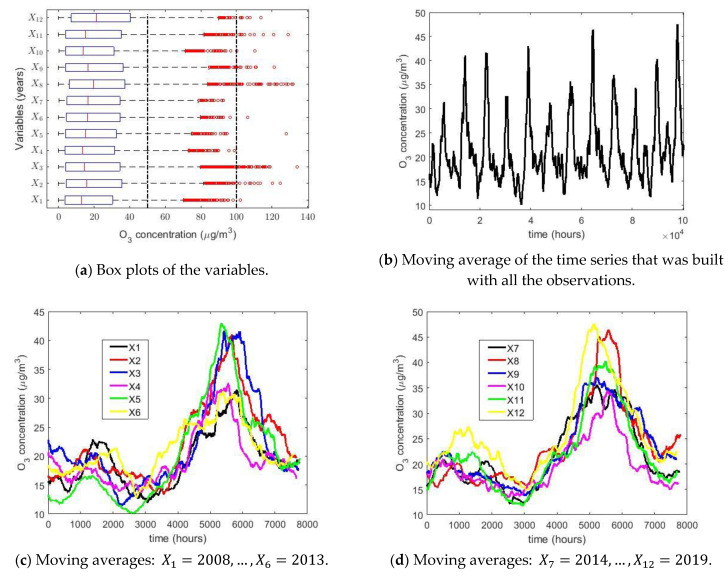
Multiple box plots of the variables (**a**) and moving averages (**b**–**d**) n the box plot diagrams, the red circles represent the outliers and, according to the IAQ of Quito [23], the vertical dashed lines indicate the following intervals of levels of air pollution in $\mu g/m^{3}$ due to O_3_ concentration in Quito: Desirable = [0, 50); Acceptable = [50, 100); and Caution = [100, 200).

In order to summarize the observations, Table 1 shows some summary statistics. In addition, Figure 2a shows a multiple box plot of the variables $X_{k}$, k=1,⋯,12. In this figure, a separation between air pollution levels due to O_3_ concentration in Quito [23] is made by using dashed vertical lines. These levels in $\mu g/m^{3}$ are as follows: Desirable = [0, 50); Acceptable = [50, 100); and Caution = [100, 200).

In all the variables shown in Figure 2a, the following comparisons can be made: mean > median, skewness > 0, and kurtosis > 2.7. Furthermore, kurtosis > 4 in both 2010 and 2015. All of the aforementioned indicates that the distributions of $X_{k}$, k=1,⋯,12, are heavy-tailed [19,21], or that these variables can be considered due to the existence of a mixture of distributions. In addition, in Figure 2a it can be seen that each year presents observations above the desirable level. Additionally, it is observed that abnormally high observations are present almost every year, although the percentage of such observations does not exceed 2.2%. Moreover, it is observed that the desirable level is always far exceeded, although the average does not exceed these values by much. All this confirms, once again, that the samples do not come from a normal distribution.

The moving averages (MAs) [77,78] of all years are shown in Figure 2b, the MAs of the years from 2008 to 2013 are shown in Figure 2c, and the MAs of the years from 2014 to 2019 are shown in Figure 2d. In this research, the size of the moving average MA was equal to 720, which represents the number of samples that can be taken in a month, assuming that each month has a duration of 30 days. The graph of MAs of all years indicates a stable concentration of O_3_. Furthermore, the maximum and minimum are reached in the third and second quarters, respectively. This corroborates the findings of [23]. Additionally, it is observed that when the observations are smoothed, none of the values fall outside the interval of desirable air pollution values. Therefore, the overcoming of this level is in specific moments and, when this occurs, it does not take place in a sustainable way.

**Figure 3:**
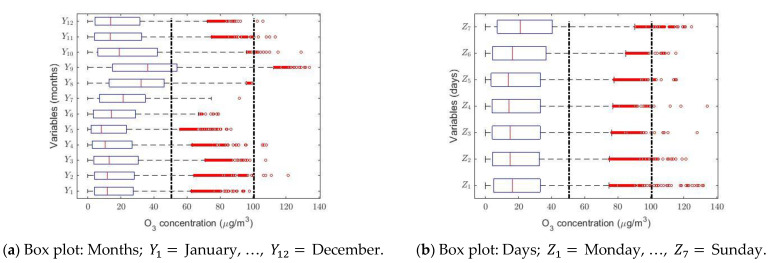

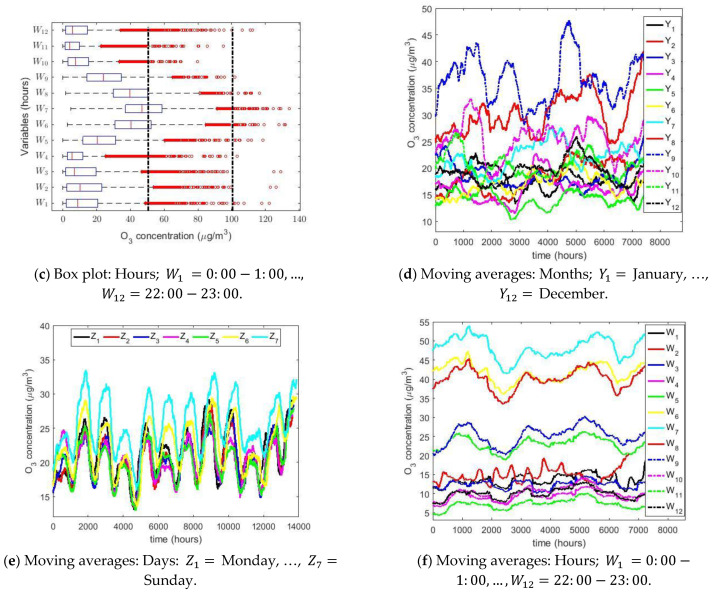
Box plots of the variables (**a**–**c**) and moving averages (**d**–**f**). In the box plot diagrams, the red circles represent the outliers and, according to the IAQ of Quito [23], the vertical dashed lines indicate the following intervals of levels of air pollution in $\mu g/m^{3}$ due to O_3_ concentration in Quito: Desirable = [0, 50); Acceptable = [50, 100); and Caution = [100, 200).

The analysis performed by months, days, and hours confirmed the previous conclusions. Figure 3a–c shows that the observations have heavy-tailed distributions. In addition, Figure 3a shows that the O_3_ concentration is lowest in the summer months, although the maximums occur in September and October. There do not appear to be any differences in O_3_ concentration when the analysis is carried out by days (see Figure 3b). Furthermore, Figure 3c shows that the O_3_ concentration grows slightly at noon and that there are a lot of abnormally high observations in all time slots.

Figure 3d–f shows that the months, days, and hours have similar behavior throughout the years. In short, if the O_3_ concentration values are higher or lower in any period of a particular year or day, something similar occurs for the rest of the years. In the case of the analysis by days (see Figure 3e), more noticeable periodic behavior appears. Moreover, it is possible that the periodic behavior observed in Figure 3e also manifests itself in Figure 3d,f. Therefore, it is likely that the samples come from a variable that presents behavior patterns that repeat every certain time interval. However, the frequencies of the possible oscillations that can be seen in Figure 3d–f seem to be neither the same nor integer multiples of the others. Additionally, it does not seem that some frequencies are rational multiples of others. Therefore, it can be said that this type of signal could have characteristics that are typical of variable frequency signals, among other things. The mathematical modeling of this type of signal belongs to different research that is not part of the objective of this paper.

In this research, different transformations of variables were made [79], because classical statistical inference methods could not be used. The previous paragraphs have shown that the data distribution was not normal. Nevertheless, this strategy did not give satisfactory results, because the data of the years could only be fitted to heavy-tailed distributions.

Furthermore, in order to analyze the ozone concentration by months, weeks, and hours, several transformations of variables were also attempted, and some transformations of a very few variables produced adjustments close to the logistic or normal distributions. However, when these changes were attempted for the rest of the variables of the same type, it was not possible to achieve distributions that were not heavy-tailed.

Therefore, the aforementioned was the motivation for using both nonparametric statistics and robust statistics to perform the type of data analysis presented in this paper.

## 4. Nonparametric Analysis

In this paper, after having found that there was almost no linear correlation between samples, it was concluded that they came from linearly independent variables. In addition, the samples were compared with each other by making use of the Wilcoxon signed-rank test (WSRT) [43]. Here, $M_{e}=M_{0}$ was the null hypothesis ($H_{0}$), where $M_{e}$ stands for the median; the alternative hypothesis $\left(H_{1}\right)$ was $M_{e}\neq M_{0}$. Specifically, if $H_{0}$ is true and observations behave in a stable manner, then half of the observed values are less than $M_{0}$.

In the case under study, at the (1−α) confidence level [43], the nonparametric confidence intervals should be as follows: $P\left(X_{\left(k_{\alpha /2}^{\prime }\right)}<M_{e}<X_{\left(k_{\alpha /2}\right)}\right)=1-\alpha$, where $k_{\alpha /2}^{\prime }=N/2+0.5-z_{\alpha /2}\sqrt{N/4}$, $k_{\alpha /2}=N/2+0.5+z_{\alpha /2}\sqrt{N/4}$, N is the sample of length, and, at (1−α)/2, $z_{\alpha /2}$, depends on the normal distribution [43,79].

**Figure 4:**
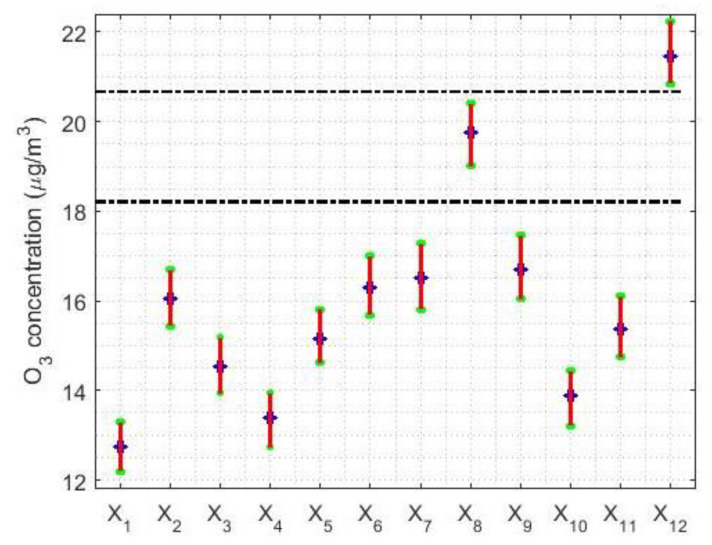
The 95% confidence intervals. Nonparametric intervals for the median: $X_{1}=2008,\;\ldots ,\;X_{12}=2019$. The separation of categories is carried out using horizontal dashed lines.

The limits of the confidence intervals are shown in Table 2, and Figure 4 shows a graphical representation of these intervals. From the analysis of Table 2 and Figure 4, we see that no trend in the concentration of O_3_ is clear. It can be seen that there were three years, 2008, 2011, and 2017, that seemed to have lower O_3_ concentrations. However, there is an overlap of intervals with all the years, with two exceptions: 2015 and 2019. These two years have higher O_3_ concentration levels than the rest of the years, especially 2019. In addition, 2015 and 2019 are different from each other. On the other hand, the widths of the confidence intervals have no significant differences. Moreover, it is verified once again that the desirable level of air pollution [23] was exceeded.

Furthermore, Figure 4 shows that the data can be grouped into three categories. One formed by the variables $X_{8}$ (corresponding to 2015), another formed by the variable $X_{12}$ (corresponding to 2019), and the third formed by the rest of the years. The WSRT [43] showed that the variables’ median homogeneity was the same as that obtained using nonparametric confidence intervals, although with some small differences because *p*-values [43] greater than 5% were considered. For example, $X_{1}$ is significantly different from the distribution of $X_{3}$, but not different from $X_{4}$. Moreover, $X_{3}$ is also not significantly different from $X_{4}$.

**Figure 5:**
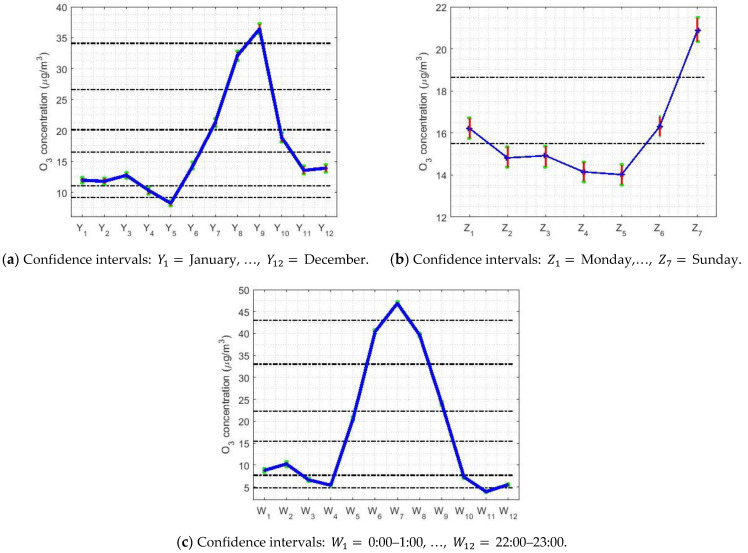
The 95% nonparametric confidence intervals for the median. The separation of categories is carried out using horizontal dashed lines.

For the nonparametric analysis of the months (see Figure 5a), seven categories were established. In this case, the use of the nonparametric intervals and the WSRT yielded the same results. According to the months, the level of O_3_ concentration seems to be a periodic signal. At the end of summer, the ozone concentration levels are the highest, when there is less activity in the city, and these levels reach their minimum value in May. In addition, a stabilization occurs between the first and last months of the year, and between May and November a rebound occurs and the stabilization level is reached. Additionally, the nonparametric confidence intervals’ amplitude was directly proportional to the value of the median.

Figure 5b shows that, when analyzing the weeks, the concentration of O_3_ increased notably on the weekends and continued to decrease on weekdays. In this case, the WSRT and the nonparametric intervals yielded the same results. Furthermore, the width of the nonparametric confidence intervals was directly proportional to the value of the median.

Finally, in the analysis of hours (see Figure 5c), the level of O_3_ concentration reached its maximum value, close to $50\;\mu g/m^{3}$, around 12:00. For the rest of the hours, the concentrations were close to $5\;\mu g/m^{3}$ around 7:00 and around 22:00. Moreover, there were more categories than for the analysis of the days of the week, because between states of high concentration and low concentration there were transition variables. Between 22:00 on the current day and 7:00 on the following day, the O_3_ concentration remained stable, with minor changes.

## 5. Robust Analysis

The analysis carried out in this section is based on robust statistics, which are not sensitive to extreme observations. As a consequence, the estimations of the data distribution and dispersion performed in this section had little sensitivity to the influence of extremely high or low observations [18,19,20]. In addition, the foremost objective of this section is to analyze the observations by using these estimations.

Furthermore, in order to prevent robust estimators from being affected by extreme observations, the influence curve [80] is used to characterize the robust statistics of this paper. Moreover, the order sample statistics [43] is used to obtain the robust estimators found in the paper.

### 5.1. Location and Scale Estimators

Location estimators [18,19,20,81]. TM: Trimean; $T_{wa}\left(c\right)$: Andrew’s wave; T(α): α-trimmed mean; $T_{bi}\left(c\right)$: Biweight; and W(α): α-winsorized mean.

Scale estimators [18,19,20,80,82,83]. $s_{x}$: Sample standard deviation; MAD: Median absolute deviation; $MAD_{mean}$: Mean absolute deviation; $s^{W}\left(\alpha \right)$: Winsorized standard error; SRH: One-half of the fourth-spread; $s_{\omega a}\left(c\right)$: Andrew’s wave; $C_{n}^{\alpha }$: Estimator based on a subrange; LMS: Least median squares; and $S_{bi}\left(c\right)$: Biweight.

**Figure 6:**
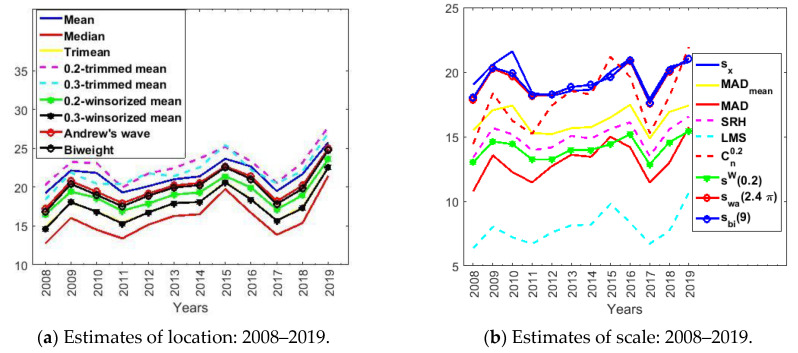
Estimates of location and scale by year.

Table 3 and Table 4 show the point estimates of location and scale, respectively. In addition, the above estimators are graphically represented in Figure 6.

In Figure 6a, according to the location estimates, the ozone concentration is stable, with two increases in 2015 and 2019; the rise occurring in 2019 is more pronounced. It is interesting to see that the 0.2-trimmed mean and the median are the boundaries of the location measures.

In Figure 6b, according to the scale estimates, the standard deviation and the *LMS* point estimator are the boundaries of the scale measures, with the biweight midvariance being very similar to the Andrew’s wave.

Likewise, it is noteworthy that the scale estimates are quite stable for each estimator and very large compared to the location estimates. These estimates indicate great variability in the O_3_ concentration measurements, which, on the one hand, have few outliers, but, on the other, have many high observations compared to the center of the distribution. What was said previously was already appreciated when analyzing Figure 2a.

**Figure 7:**
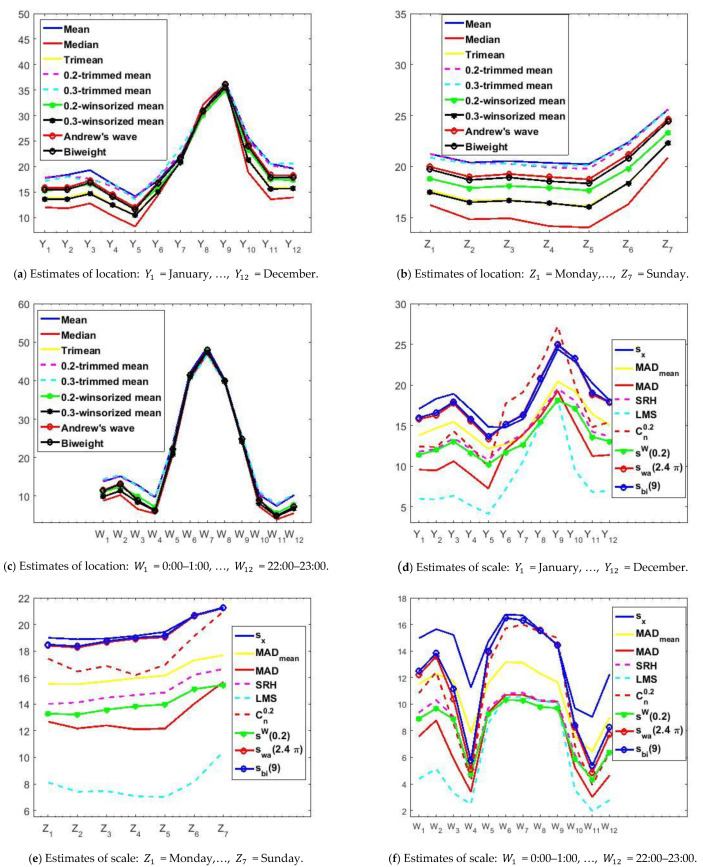
Estimates of location and scale: month, weeks, and hours.

So that the O_3_ concentration analysis could also be carried out over periods of time that are representative of the daily activity of human beings, we decided to repeat what was done previously for the analysis of the years, but this time to analyze the O_3_ concentration by months, days, and hours. Figure 7 shows graphical representations of the new estimates.

The location estimates of the variables of the months (see Figure 7a) are all very similar to each other; the higher the value of the estimate, the more similar these estimates are. Additionally, the scale estimates are very stable (see Figure 7d). The boundaries of these estimates are $s_{X}$ and $C_{n}^{0.2}$ (upper boundary) and the *LMS* point estimator (lower boundary). Furthermore, despite having few outliers, the variability is high because the value of the scale estimates cannot be discarded.

Figure 7b shows that the O_3_ concentrations increase on the weekends, keeping the location estimates stationary. The O_3_ concentration during the weekend is higher than during the first days of the week. All estimates are bounded by the median and the mean.

Figure 7e shows that the scale estimates evolve in parallel in a band of values. It is notable that the lower bound of all these estimates are the estimates given by the *LMS* point estimator, just as it happens in Figure 7d,f. In other words, the *LMS* scale estimates are much lower than the other estimates. Furthermore, the biweight, standard deviation, and Andrew’s wave values are very high. This result suggests that there is high variability without the need for extreme observations to be all outliers because the biweight and the Andrew’s wave are robust.

In the day location estimates (see Figure 7c), from 7:00 there is a very abrupt rise in the concentration of O_3_ until noon, going from $5\;\mu g/m^{3}$ to about $48\;\mu g/m^{3}$. Afterward, there is also a very pronounced drop until 20:00, which is when the commercial activity ends. For the rest of the hours, the O_3_ concentration is similar, remaining at very low levels.

Regarding the scale estimates of the hours of the day (see Figure 7f), these estimates have a behavior similar to that of the other variables. In short, the boundaries of these estimates are the standard deviation and the *LMS* estimator. In general, these scale estimates appear to differ from each other in a constant and the increase is related to the increase in the location estimate.

Finally, it is worth mentioning that the same pattern seems to be followed by the months, weeks, and hours. We observed that, for each variable, the curves grew and decreased at the same time. Furthermore, we observed that the more the ozone concentration increased, the more the variability increased, and vice versa.

### 5.2. Confidence Intervals

In this research, the confidence intervals were built using the methodology explained in [48,49,50], which is in accordance with [84,85]. These intervals allowed us to perform the variable classification and categorization and recognize similarities and differences. The intervals are as follows:

- $\left(\bar{X},s_{x}\right)$, where $\bar{X}$ is the mean
- $\left(M_{e},\;MAD\right)$, where $M_{e}$ is the median
- $\left(M_{e},\;IQR\right)$, where IQR is the interquartile range
- $\left(T\left(\alpha \right),s^{W}\left(\alpha \right)\right)$
- $\left(T_{wa}\left(c\right),s_{wa}\left(c\right)\right)$
- $\left(T_{bi}\left(c\right),s_{bi}\left(c\right)\right)$.

**Figure 8:**
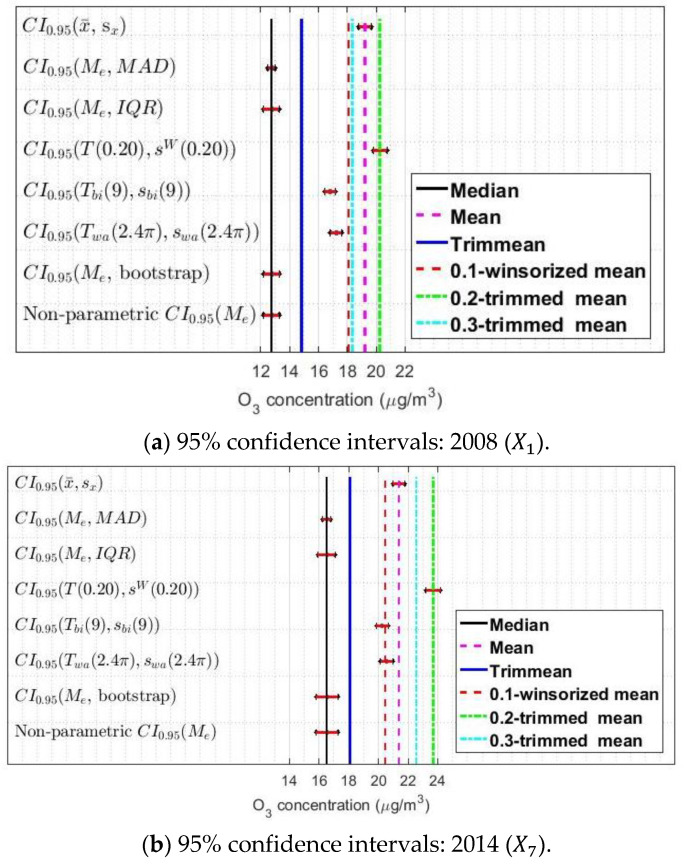

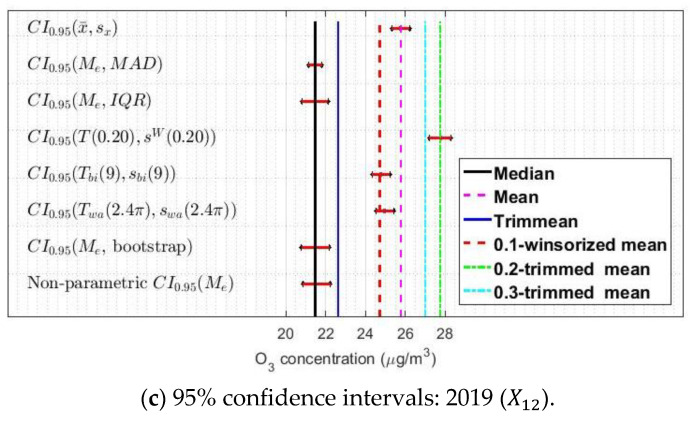
The 95% confidence intervals ($CI_{0.95}$): 2008 $\left(X_{1}\right)$, 2014 $\left(X_{7}\right)$, and 2019 $\left(X_{12}\right)$.

In addition, other confidence intervals were built by using a bootstrap method [20,49,50]. As a result, eight confidence intervals were built: one classic, one bootstrap, one parametric, and five robust. The intervals have been included in this paper for three of the 12 variables under study. Specifically, intervals are shown here for 2008, 2014, and 2019 (see Figure 8). That is, the intervals are shown for the two end variables and one intermediate. Showing these intervals for more variables would not contribute significantly to the analysis carried out in this section.

Analyzing Figure 8, it can be concluded that all the variables have similar characteristics. First, the 0.2-trimmed mean and winsorized variance-based confidence intervals are the most shifted to high concentrations, closely followed by the classic confidence intervals. The difference is approximately $2\;\mu g/m^{3}$. Second, the biweight confidence interval is very similar to the Andrew’s wave confidence interval. Third, the intervals with the lowest values are those based on the median. Furthermore, a similarity between nonparametric and bootstrap intervals is observed. Additionally, the pair ($M_{e},\;MAD$) yields the narrowest confidence intervals. Finally, it should be noted that there is a slight increase in estimates for the early years compared to later years.

According to what has been previously said, the estimators $\left(T\left(\alpha \right),s^{W}\left(\alpha \right)\right)$ and $\left(T_{bi}\left(c\right),s_{bi}\left(c\right)\right)$ were used in this paper to compare the variables under study, for the following reasons:

1. Classic confidence intervals are based on the fact that an approximately normal underlying distribution is assumed. However, in this research that is not the case.
2. The $\left(M_{e},\;MAD\right)$ and $\left(M_{e},\;IQR\right)$ point estimators, the bootstrap estimators, and the nonparametric estimators give similar results. Nevertheless, when groupings of variables are analyzed, the pair $\left(M_{e},\;MAD\right)$ allows for establishing a greater number of differences between them.
3. The estimators based on the biweight and Andrew’s wave yield similar results. Therefore, it is acceptable to use one type of estimator or the other to carry out the comparative analysis.

**Figure 9:**
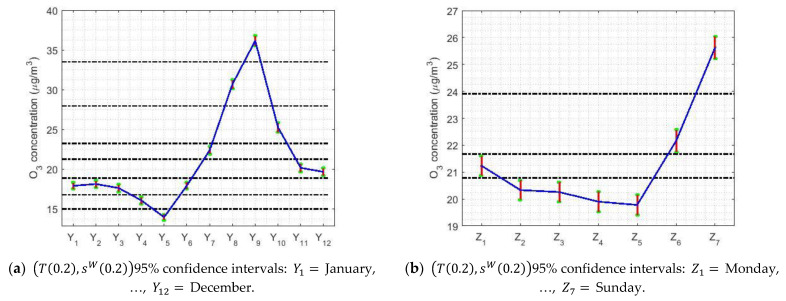
The 95% confidence intervals: $X_{1}=2008,\;X_{2}=2009,\ldots ,\;X_{12}=2019$. The separation between categories is made by the horizontal dashed lines.

Information on the confidence intervals for the pair of estimators $\left(T\left(0.2\right),s^{W}\left(0.2\right)\right)$ and $\left(T_{bi}\left(9\right),s_{bi}\left(9\right)\right)$ is given in Table 5. In addition, graphical representations of these intervals are shown in Figure 9. Moreover, horizontal lines have been used in Figure 9 to carry out the classification of the variables, which is the same as that performed in Section 4. The only difference between the classification obtained in this section and the classification obtained using nonparametric estimators (see Section 4), is that the variables $X_{1}$, $X_{4}$, and $X_{10}$ can be grouped in the same category for the pair of estimators $\left(T\left(0.2\right),s^{W}\left(0.2\right)\right)$ and for the pair of estimators $\left(T_{bi}\left(9\right),s_{bi}\left(9\right)\right)$ in a clearer way.

Regarding the amplitudes of these confidence intervals, it can be said that the pair $\left(T\left(0.2\right),s^{W}\left(0.2\right)\right)$ yielded confidence intervals that are approximately 15% wider than those obtained with the pair $\left(T_{bi}\left(9\right),s_{bi}\left(9\right)\right)$. In addition, the median and amplitude of these intervals evolve in parallel.

**Figure 10:**
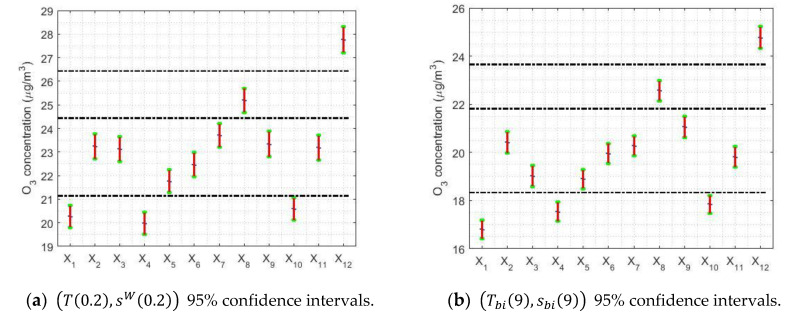

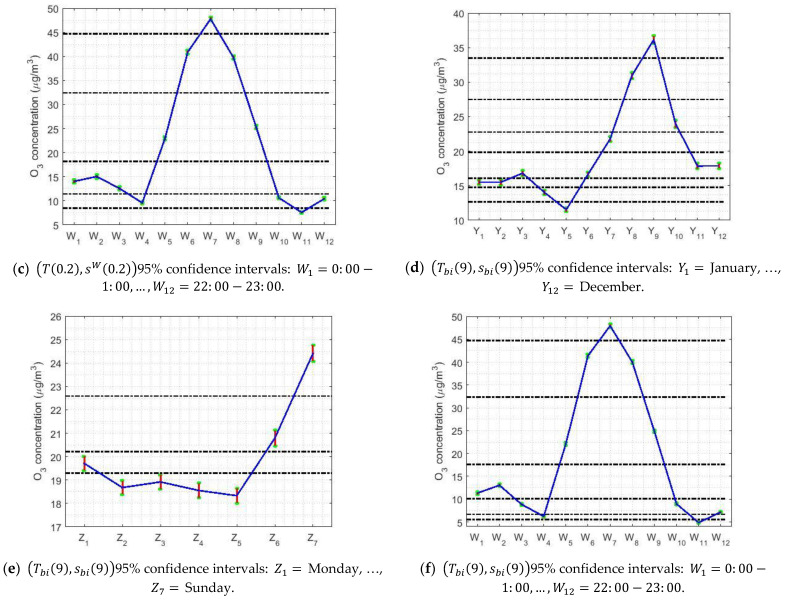
The 95% confidence intervals. The separation between categories is made by the horizontal dashed lines.

Figure 10 shows graphs that are used to study confidence intervals. In this case, the variables are months, days, and hours. In Figure 10, 95% robust confidence intervals have been found, on the one hand, using the α-trimmed mean location estimator and the $s^{W}\left(\alpha \right)$ scale estimator; and, on the other hand, using the biweight estimators $\left(T_{bi}\left(c\right),s_{bi}\left(c\right)\right)$.

In Figure 10a,d, it can be seen that May is when the lowest values (around $12\;\mu g/m^{3}$) are reached and the maximum (around $38\;\mu g/m^{3}$) is reached in September. This shows that the ozone concentration triples in the summer.

On the other hand, the descent seems to present two steps, one reached in November, when it drops by half (approximately $18\;\mu g/m^{3}$), and the other that until March remains stable and then falls until May. Figure 5a shows similar results. In addition, it seems that the analysis of the years yielded wider confidence intervals. Furthermore, it is observed that the median and the width of the intervals are directly related.

Figure 10b,e shows that the analysis for the weeks is in every way analogous to that in Figure 5b, using nonparametric estimators. In short, the maximum is reached on weekends and the values on working days are 33% lower than on weekends. Moreover, there are four categories: (I) Saturday; (II) Sunday; (III) Monday; and (IV) Tuesday, Wednesday, Thursday, and Friday. It is worth mentioning that Mondays and Saturdays are transition categories between working days and Sunday.

Furthermore, Figure 10c,f shows that the analysis for the hours is the same as the one using nonparametric estimators (see Figure 5c). In this case, the maximum is reached at approximately noon, at the moment that most solar lighting occurs, and these confidence intervals have very steep falls that reach two relative minimums. One minimum is around 7:00 and the other is around 21:00, with the deepest fall being seen in this last hour.

It is important to observe in Figure 10c,f that the O_3_ concentration goes from approximately $48\;\mu g/m^{3}$ to approximately $4\;\mu g/m^{3}$, which is a 12-fold reduction. In addition, the O_3_ concentration remains sustained between narrow limits between 21:00 on one day and 6:00 on the next.

Finally, the difference between the pairs of estimators $\left(T\left(0.2\right),s^{W}\left(0.2\right)\right)$ and $\left(T_{bi}\left(9\right),s_{bi}\left(9\right)\right)$ is that the separation level of the categories that is achieved by using $\left(T_{bi}\left(9\right),s_{bi}\left(9\right)\right)$ is better than that achieved using $\left(T\left(0.2\right),s^{W}\left(0.2\right)\right)$.

### 5.3. Data Transformation of the Hours at Which the O_3_ Concentration Is the Highest to Make Them Fit a Normal Distribution: Analysis by Months and Days of the Week

Sometimes it is important to optimize the data sampling process because there are some applications in which researchers are interested in reduced power consumption when they are using portable devices powered by a battery. This is why, in this part of the paper, some of the categories that have already been established for the months, days, and hours in previous sections are going to be grouped into categories that represent the hours of the highest O_3_ concentration.

For the station under study, the analysis carried out previously revealed that the hours of the day at which the ozone concentration levels are highest are between 10:00 and 15:00 (that is, the central hours of the day). Therefore, here we have tried to fit the variables that represent these hours by means of transformations to a normal distribution (that is, $W_{6}$ = 10:00–11:00, $W_{7}$ = 12:00–13:00, and $W_{8}$ = 14:00–15:00). The aforementioned is extremely important because if the desired fittings are achieved, it is possible to use classical statistical inferences to obtain confidence intervals for the mean. Once this is done, the variable changes made can also be undone to obtain confidence intervals for measures of location of the original variables.

**Figure 11:**
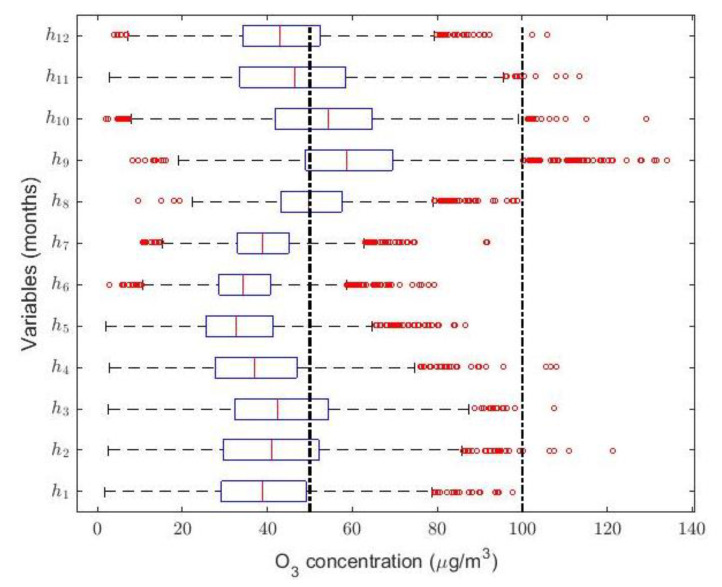
Box plot diagram of the variables $h_{i}$, i=1, …, 12. The red circles represent the outliers and, according to the IAQ of Quito [23], vertical dashed lines have been used to establish the separation between O_3_ concentration levels in $\mu g/m^{3}$: Desirable (i.e., [0, 50)), Acceptable (i.e., [50, 100)), and Caution (i.e., [100, 200)).

In order to do the above, the variables $h_{i}$, i=1,…,12, have been introduced to represent the O_3_ concentration in the time interval 10:00–15:00 in each of the months. That is, $h_{1}$ represents the abovementioned concentration in January, $h_{2}$ represents the abovementioned concentration in February, and so on. Table 6 shows the coefficient of skewness of the variables $h_{i}$, i=1,…,12, and Figure 11 shows their box plot.

Given the shape of the distributions, we used different transformations of variables [68]: linear, logarithmic, and inverse, among others. However, the transformations that have given the best results have been those of the form: $v_{i}={\left(a\cdot h_{i}+b\right)}^{q}$, i=1,…,12, where a, b and q are positive real numbers. Statistical information on the transformations made to the variables under study is given in Table 7.

Therefore, taking into account the information on the *p*-value shown in Table 7, all transformations have been carried out at a 0.01 level of significance. In other words, this means that there is a 1 in 100 chance that we would reject the hypothesis that the transformed variable is Gaussian when it should be accepted. However, despite the fact that transformations to normal distributions have been achieved, there is no pattern that could serve to describe a vast majority of the variables considered. In reality, the process of obtaining the transformation of the variable that can give rise to a normal distribution can be quite laborious, and it is not guaranteed that the desired results will be obtained.

Next, it will be compared whether, once the appropriate transformation has been found, there are significant differences between the classical intervals obtained after the change of variable and those obtained using robust statistics.

**Figure 12:**
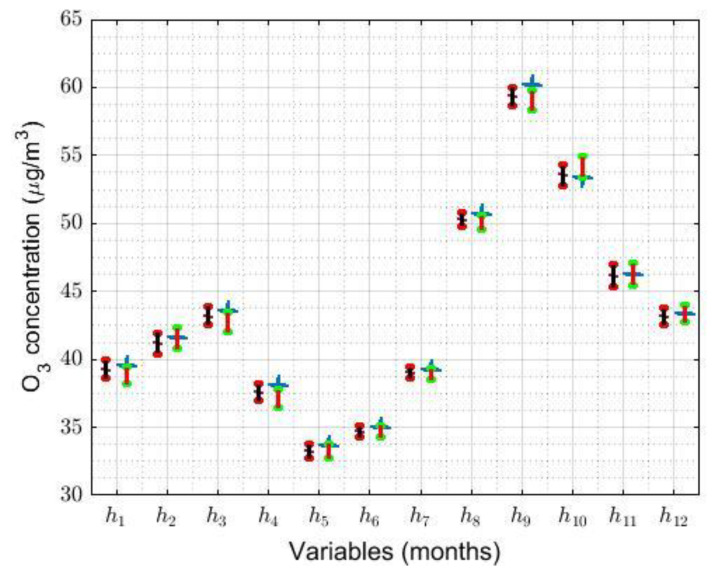
The 95% robust and classic confidence intervals. The robust confidence interval (in black) and robust centralization estimator (at the center of the robust interval) are shown on the left-hand side of $h_{i}$, i=1, …, 12. In addition, for the mean of the transformed variable (i.e., $v_{i}$, i=1, …, 12), the inverse transformation of the interval is shown on the right-hand side of $h_{i}$ (in red). In the *i*-th classic interval, the mean of the variable $h_{i}$ is represented by an x (in blue).

The robust confidence intervals and robust centralization estimators, based on the biweight estimators $\left(T_{bi}\left(9\right),s_{bi}\left(9\right)\right)$, are represented in Figure 12 to the left of each of the original variables, $h_{i}$, i=1,…,12 (on the abscissa axis). Likewise, for the mean of the adjusted normal distribution, the inverse confidence interval is shown on the right side of the original variables, together with the mean of these variables.

Figure 12 shows that both confidence intervals (i.e., the robust confidence interval and the classic confidence interval) are very similar, in terms of both their location and their length. Furthermore, in the case of the variables $h_{4}$ and $h_{9}$, the mean is not included within the classical intervals. This shows that the number of outliers makes the distributions of the original variables have heavier tails than the normal distribution. Moreover, as in the other variables (i.e., $h_{1},\ldots ,h_{3},\;h_{5},\ldots ,h_{8},h_{10},\ldots ,h_{12}$) the mean falls within the classic confidence interval, so the desired estimate can be found by using this type of confidence interval.

**Figure 13:**
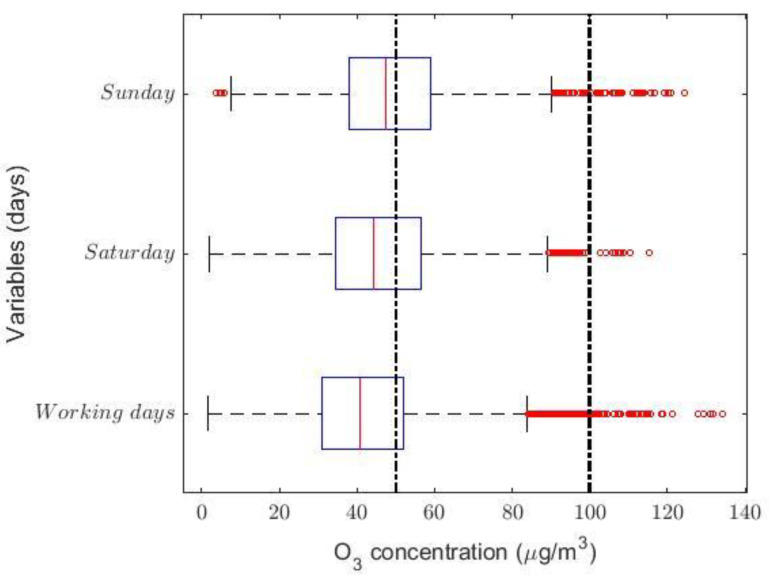
Box plot diagram of the variables *Working days*, *Saturday*, and *Sunday*. The red circles represent the outliers and, according to the IAQ of Quito [23], vertical dashed lines have been used to establish the separation between O_3_ concentration levels in $\mu g/m^{3}$: Desirable (i.e., [0, 50)), Acceptable (i.e., [50, 100)), and Caution (i.e., [100, 200)).

In order to conclude the study, an analysis similar to that performed previously will be carried out below, but now said analysis is aimed at studying the hours with the highest O_3_ concentration. Therefore, this new analysis will be carried out in the same time slot used previously, depending on the day of the week and distinguishing between working days (i.e., Monday to Friday) and weekends. These three new variables (i.e., *Working days*, *Saturday*, and *Sunday*) have been chosen, because Sunday is the day of the week on which there is the highest O_3_ concentration, and Saturday behaves as a transition period between the days people normally have to work and Sunday. The skewness coefficient of the variables *Working days*, *Saturday*, and *Sunday* is shown in Table 8, and their box plot is shown in Figure 13.

In Figure 13, it is observed that the O_3_ concentration grows when going from working days to Sunday and that Saturday is a transition category. Moreover, it is observed that these variables seem to have come from heavy-tailed distributions, which can be inferred from the values shown in Table 8 and the box plot diagrams shown in Figure 13. Figure 9 shows statistical information on the transformations made to the variables to make them fit a normal distribution. The transformed variables are shown in Table 9 and all transformations have been carried out at a 0.05 level of significance.

When grouping the hours and working days, it turns out that the change of variable is quite drastic. Furthermore, Table 9 shows that the *p*-value of the fit of the working days to a normal distribution is the lowest of the three. Additionally, the grouping of the days with a rather artificial, far-fetched transformation, $u_{1}$, and a low *p*-value is an indicator that a combination of different statistical populations is being analyzed.

If a far-fetched transformation and a low *p*-value indicate the existence of a mixture of distributions, then the variables $v_{6}$, $v_{7}$, and $v_{9}$ (see Table 7) tell us that the months of June ($h_{6}$), July ($h_{7}$), and September ($h_{9}$) could be analyzed for weeks or fortnights. However, if this is the case, researchers should again start looking for a transformation similar to a normal distribution; then they would have to find the confidence intervals for the mean and, finally, they would have to undo the change of variable. In short, the whole process would have to be repeated again, including what was done up to Section 5.2.

**Figure 14:**
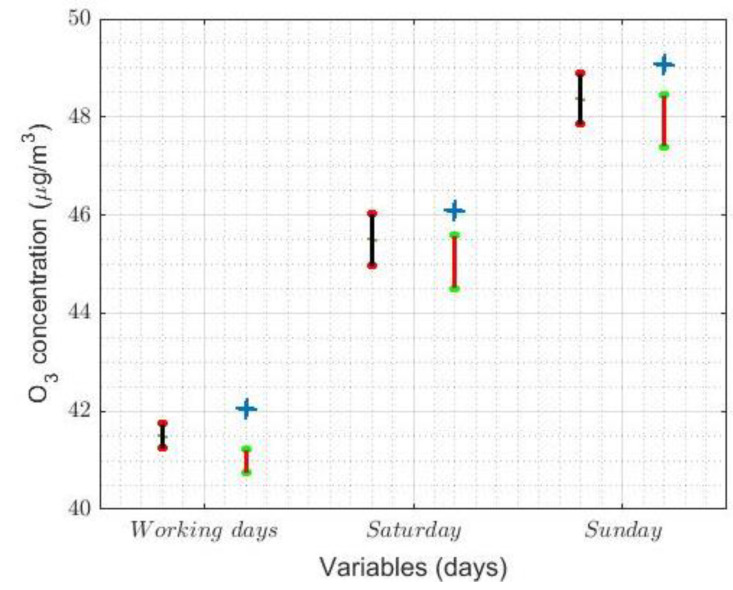
The 95% robust and classic confidence intervals. The robust confidence interval (in black) and robust centralization estimator (at the center of the robust interval) are shown on the left of the variables: *Working days*, *Saturday*, and *Sunday*. Additionally, for the mean of the transformed variable (i.e., $u_{i}$, i=1, 2, 3), the inverse transformation of the interval is shown on the right of each untransformed variable (in red). Above the *i*-th classic interval, the mean of the untransformed variables is represented by an x (in blue).

As in Figure 12, Figure 14 shows the robust confidence intervals and robust centralization estimators, based on the biweight estimators $\left(T_{bi}\left(9\right),s_{bi}\left(9\right)\right)$, to the left of each of the original variables. On the other hand, for the mean of the adjusted normal distribution, the inverse transformations of the confidence intervals are represented on the right-hand side of the original variables, together with the mean of these variables.

In this case, both types of confidence intervals (i.e., classic and robust ones) are similar in length and, in the case of Saturday and Sunday, they have a nonzero intersection. On the other hand, in the case of working days, the intersection of these intervals is zero, but these intervals do not touch for less than $0.1\;\mu g/m^{3}$. Additionally, the mean is not included in any of the classic confidence intervals. Therefore, in this case, the classic confidence intervals are not useful to find the desired estimate.

In view of the results, the following conclusions can be drawn: Obtaining confidence intervals based on robust estimators has turned out to be more efficient than building classic confidence intervals obtained by transforming the variables. As has been shown, in order to obtain classical confidence intervals, transformations of variables must be sought that make them fit a normal distribution. However, these transformations are not homogeneous for all variables and, in some cases, admissible transformations are not obtained either. In fact, in terms of statistics, several of the obtained transformations are rare, artificial, and far-fetched.

Furthermore, something very important that must be highlighted is that, after having done everything that has been done in this subsection (i.e., in Section 5.3), the results obtained in the best of the studied cases are analogous to those previously obtained from the robust estimators of the data provided by the primitive measurements.

## 6. Discussion

A preliminary analysis of the observations yielded that the ozone concentration at Belisario is not a threat to human beings. Most of the samples are at an air pollution level that is considered to be acceptable [23]. Only in specific cases is the level of caution reached, and it happens in a nonsustained manner.

Nevertheless, it should not be forgotten that each city establishes its own criteria for air quality, and what may be acceptable in some cities may not be acceptable in others. However, the truth is that they all share a common goal: to improve the air quality that citizens who live in them breathe every day.

As has happened in previous studies [48,49,50,51], the variables chosen for the analysis present a large number of outliers. Therefore, this could indicate that these variables come from heavy-tailed distributions. The foregoing justified the need to combine different types of analyses, in order to explain precisely how the ozone concentration has behaved at the station under study from 2008 to 2019.

The data smoothing process showed a tendency for the O_3_ concentration to be entrenched in a range of values. Likewise, for the months, a notable maximum occurred at the end of the summer and, for the hours of the day, the maximum occurred at noon. In this case, we observed notable reductions to the right and left; the concentration of O_3_ was then maintained at a stable level until a new rise began. In contrast, the differences between days of the week were seen between weekends and working days, although the ups and downs were not very pronounced.

Once all the previous analyses had been carried out, the authors tried to make the variables under study fit a normal distribution by using variable transformations, because the classic inferential analysis was attempted to be applied. However, the obtained results were not as expected, because when a parametric distribution was attained using a variable transformation for a particular time period, an acceptable fit was not achieved for the other periods. Therefore, the techniques used to carry out the analysis had to be nonparametric and robust.

Here, it was possible to build different groups of variables by using the WSRT. Moreover, three strata were established to analyze the years by using nonparametric methods. These strata were as follows: one stratum formed by 2019, another by 2015, and the third by the other years. Nevertheless, in the last stratum 2008, 2011, and 2017 can be separated, because these years have the lowest levels of O_3_ concentration.

Regarding the analysis carried out for the months using nonparametric methods, seven classes were obtained. However, they were then decreased to four.

Regarding the nonparametric analysis of the days, they were grouped into the last four working days of the week and Monday, Saturday, and Sunday, with Sunday forming one class and Monday and Saturday forming another class. The reason for this classification is the rise in the O_3_ concentration on weekends as opposed to working days, and Monday and Saturday being transition categories.

Regarding the hours of the day, many groupings were obtained using nonparametric methods. In this case, a maximum was obtained at noon, the transition categories between the maximum and the minimum corresponded to the hours from 8:00 to 20:00, and in the rest of the hours, from 21:00 on the present day to 7:00 the following day, the O_3_ concentration remained fairly unchanged.

Next, robust confidence intervals were built by using location and scale statistics that are robust, because they are highly immune to the influence of extreme observations. The analysis carried out using these confidence intervals showed that the concentration of ozone stayed stable in the range of desirable air pollution level [23], decreasing in 2008, 2011, and 2017, and increasing in 2015 and 2019, with the last year being the one in which the concentration rose much more.

The analysis showed a parallel between location and scale estimates. In short, an increase in the value of the location estimate produced an increase in the value of the scale estimate. One conclusion that can be drawn from this is that the location and scale estimates have been determined by the outliers.

The robust analysis based on confidence intervals allowed us to observe that, in terms of years, a maximum occurred in 2015 and a more pronounced maximum occurred in 2019. In addition, in a more notable way than with the nonparametric analysis, with the robust analysis, 2008, 2011, and 2017 created the lowest value category. Moreover, there were more categories in which the variables were classified when robust intervals are used, because the robust confidence intervals were narrower than the nonparametric ones.

Furthermore, a certain periodicity was observed for the variables corresponding to the months, days, and hours. The analysis of the months allowed us to observe notable increases at the end of the summer, decreases in May and October, and stable behavior in both the first quarter of the year and the last months of it.

The analysis of days allowed us to observe an appreciable difference between the O_3_ concentration on working days and the O_3_ concentration at weekends. Lastly, in the analysis of the hours of the day, minimum values were observed at 7:00 and 21:00. Additionally, it was observed that the maximum occurred at noon, the transition values were between 7:00 and 21:00, and stable O_3_ concentrations were obtained at the rest of the hours.

The existence of maximum and minimum values in the concentration of air pollutants has also been observed in other research [50,51,86]. The reality is that anthropogenic emissions have a great influence on the levels of air pollutants in urban cities.

Other air pollution variables have been shown to have possible periodic behavior [50,51]. Nevertheless, everything seems to indicate that the way in which the data are represented has a great influence on either the appreciation or not of said periodic behavior. The in-depth mathematical modeling of the air pollution variable analyzed in this research is not the objective of this paper. However, this is work to be carried out in future research. The contribution of the present research is that it has been shown that this periodic behavior can be represented by using 95% confidence intervals, both robust and nonrobust ones. Furthermore, the range of possible values of these signals has been categorized with robust statistical precision, which is in agreement with what was discovered in [50,51].

Through all these analyses, groupings of variables were obtained and differences between categories were found. Here, the quantification of such differences was performed by using confidence intervals.

Finally, it is worth mentioning one more contribution to this paper. The possibility of forming new variables has been analyzed based on the hours of the day that have been shown to have the highest ozone concentrations. Doing this is very important because it allows researchers to optimize the data collection time and therefore save energy when using battery-powered portable measurement equipment. However, the analysis was complex because the new variables formed are different. Therefore, it is difficult to establish predictions since the variables do not come from the same statistical populations. Furthermore, using robust analysis methods was shown to be much more effective than attempting to carry out variable transformations. Here, it was shown that, after having made the variable transformations, the results were analogous at best to those that had already been obtained with the robust methods.

Another thing to keep in mind is that several of the variable transformations achieved are rare, artificial, far-fetched, or unrealistic and therefore do not clarify what the variable does. In addition, the researcher has to first do the robust analysis and then form new variables by grouping together the variables with the highest O_3_ concentration values. Therefore, it is not worth doing these additional transformations of variables if the previous robust analysis already allows for obtaining significant results. In other words, it is not advisable to form new variables after having done the robust analysis, if the new results are analogous, in the best of cases, to those already obtained with the robust methods.

## 7. Conclusions

A robust analysis of ozone concentration was conducted in this paper. This analysis was performed at Belisario station for measurements taken from 1 January 2008 to 31 December 2019. This monitoring station is in Quito, Ecuador, and the set of measurements was transformed into samples taken from several independent variables. The information from these variables was then classified and categorized. In addition, similarities and differences between variables were established with robust statistical precision.

The raison d’être of this study was to group the measurements into variables that allowed us to describe the behavior of the O_3_ concentration in a more realistic manner. As a matter of fact, people who live in cities are worried about air pollution in terms of hours, weeks, and years, and they need precise information to keep themselves and their relatives safe. The research presented in this paper can be used to know and predict how the O_3_ concentration behaves at Belisario station.

In a previous report [23], some general statements about tropospheric ozone levels at Belisario station were made; for the descriptive analysis, the only statistical tools used in [23] were the mean and maximum values. In addition, the report made reference to the main air pollution sources in Quito and presented general environmental issues. However, such a report lacks a robust, rigorous analysis that allows us to study the ozone concentration in a comprehensive manner. Therefore, one possible application of the research presented in this paper is that it can help us to study ozone concentration measurements in a statistical manner and establish robust analysis procedures.

An important fact to highlight is that the city of Quito has greatly progressed in recent years in terms of caring for the environment and improving air quality. However, much remains to be done. The authors of [47] dedicated a few words to this and gave multiple examples of things that still need to be done to improve the air quality in some areas of the city. Moreover, in [50,51] several recommendations were made which, in conjunction with those made in [47], suggested ways to decrease the level of pollution in Quito. The goal is to inform people about pollution levels in built-up areas and ensure that these pollution levels are low and not harmful to the health of human beings.

The aforementioned entails investment by urban decision-makers, research centers, universities, and so on in measuring equipment that can be located at areas of interest or is portable and battery-powered. Therefore, in order to optimize the sampling process to reduce power consumption, in this research, variables that represented the hours of the day with the highest O_3_ concentration were created. These new variables were studied and the results confirmed that, in some cases, it is essential to use robust methods to optimize the data sampling process.

Finally, for some of the variables studied, behaviors that could imply a certain periodicity were observed. However, when the researchers changed the way the data were represented by using different partitions of the time domain, these behaviors were not appreciated. Carrying out the in-depth mathematical analysis of these behaviors was not the objective of this paper, but it is something to consider in future research.

## Figures and Tables

**Table 1:** Summary statistics: ozone observations.

Year (Variable)	Count	Mean of the Variable $\left(\mu g/m^{3}\right)$	Median of the Variable $\left(\mu g/m^{3}\right)$	Standard Deviation of the Variable $\left(\mu g/m^{3}\right)$	Skewness of the Variable	Kurtosis of the Variable	Minimum Value of the Variable $\left(\mu g/m^{3}\right)$	Maximum Value of the Variable $\left(\mu g/m^{3}\right)$	Outliers of the Variable (%)
$2008\;\left(X_{1}\right)$	8741	19.2240	12.740	19.0235	1.1341	3.6247	0	102.12	1.60
$2009\;\left(X_{2}\right)$	8371	22.1576	16.050	20.5857	0.9860	3.4136	0	124.54	0.79
$2010\;\left(X_{3}\right)$	8447	21.8439	14.520	21.6142	1.2647	4.3375	0	134.08	2.12
$2011\;\left(X_{4}\right)$	8392	19.3263	13.400	18.3904	0.9742	3.1587	0	98.89	0.54
$2012\;\left(X_{5}\right)$	8484	20.1288	15.160	18.1590	0.8699	3.0665	0	128.08	0.40
$2013\;\left(X_{6}\right)$	8277	21.0235	16.290	18.5324	0.7868	2.8418	0	106.31	0.28
$2014\;\left(X_{7}\right)$	8506	21.3930	16.515	18.6472	0.7817	2.7286	0.13	92.57	0.24
$2015\;\left(X_{8}\right)$	8509	23.6667	19.770	20.0291	0.9794	4.1337	0.04	131.68	0.92
$2016\;\left(X_{9}\right)$	8384	22.6818	16.700	21.0013	0.9370	3.1916	0	121.22	0.63
$2017\;\left(X_{10}\right)$	8448	19.5031	13.875	17.9236	1.0155	3.4485	0.34	110.46	0.90
$2018\;\left(X_{11}\right)$	8405	21.6929	15.380	20.4016	0.9974	3.3810	0	129.17	0.76
$2019\;\left(X_{12}\right)$	8413	25.7776	21.470	20.7732	0.7496	2.8240	0	113.89	0.32
Total	101,107	21.5345	15.940	19.7131	0.9832	3.4724	0	134.08	0.80

**Table 2:** The 95% confidence intervals $\left(CI_{0.95}\right)$: median.

Variable (Year)	$\mathrm{Lower}\;\mathrm{Limit}\;\mathrm{of}\;\mathrm{the}\;CI_{0.95}$ $\left(\mu g/m^{3}\right)$	$\mathrm{Upper}\;\mathrm{Limit}\;\mathrm{of}\;\mathrm{the}\;CI_{0.95}$ $\left(\mu g/m^{3}\right)$
$X_{1}\;\left(2008\right)$	12.20	13.29
$X_{2}\;\left(2009\right)$	15.44	16.69
$X_{3}\;\left(2010\right)$	13.95	15.18
$X_{4}\;\left(2011\right)$	12.74	13.95
$X_{5}\;\left(2012\right)$	14.62	15.81
$X_{6}\;\left(2013\right)$	15.69	17.00
$X_{7}\;\left(2014\right)$	15.81	17.28
$X_{8}\;\left(2015\right)$	19.02	20.41
$X_{9}\;\left(2016\right)$	16.05	17.47
$X_{10}\;\left(2017\right)$	13.22	14.44
$X_{11}\;\left(2018\right)$	14.75	16.10
$X_{12}\;\left(2019\right)$	20.85	22.24

**Table 3:** Estimates of the location of the observations: point estimates $\left(\mu g/m^{3}\right)$.

Year (Variable)	Mean	$M_{e}$	TM	T(0.2)	T(0.3)	W(0.2)	W(0.3)	$T_{wa}\left(2.4\pi \right)$	$T_{bi}\left(9\right)$
$2008\;\left(X_{1}\right)$	19.2240	12.7400	14.8475	20.2708	18.3577	16.4755	14.5822	17.2095	16.7942
$2009\;\left(X_{2}\right)$	22.1576	16.0500	17.9700	23.2451	21.8878	19.4422	18.0712	20.7875	20.4229
$2010\;\left(X_{3}\right)$	21.8439	14.5200	16.9025	23.1257	20.4529	18.6331	16.8467	19.4255	19.0174
$2011\;\left(X_{4}\right)$	19.3263	13.4000	15.4825	19.9706	20.4038	16.9701	15.3045	17.9404	17.5358
$2012\;\left(X_{5}\right)$	20.1288	15.1600	16.6900	21.7689	21.8030	17.8895	16.7320	19.1783	18.8911
$2013\;\left(X_{6}\right)$	21.0235	16.2900	17.9100	22.4651	21.4295	19.0371	17.9505	20.2210	19.9591
$2014\;\left(X_{7}\right)$	21.3930	16.5150	18.0950	23.7089	22.5498	19.3308	18.0997	20.5514	20.2711
$2015\;\left(X_{8}\right)$	23.6667	19.7700	20.7250	25.1945	25.4667	21.4437	20.6082	22.7003	22.5663
$2016\;\left(X_{9}\right)$	22.6818	16.7000	18.4675	23.3377	22.2582	19.9581	18.4120	21.4043	21.0557
$2017\;\left(X_{10}\right)$	19.5031	13.8750	15.7275	20.5899	20.5212	17.1472	15.6978	18.1965	17.8385
$2018\;\left(X_{11}\right)$	21.6929	15.3800	17.4650	23.1852	22.0576	18.9715	17.2909	20.2167	19.8159
$2019\;\left(X_{12}\right)$	25.7776	21.4700	22.6300	27.7630	26.9957	23.5995	22.5443	24.9686	24.7720
All years	21.5345	15.9400	17.7425	21.3231	21.3523	19.0408	17.6475	20.2792	19.9625

**Table 4:** Estimates of the scale of the observations: point estimates $\left(\mu g/m^{3}\right)$.

Year (Variable)	$s_{x}$	$MAD_{mean}$	MAD	SRH	LMS	$s^{W}\left(0.2\right)$	$s_{wa}\left(2.4\pi \right)$	$s_{bi}\left(9\right)$	$C_{n}^{0.2}$
$2008\;\left(X_{1}\right)$	19.0235	15.5054	10.780	13.385	6.370	13.0238	17.8466	18.0195	14.4307
$2009\;\left(X_{2}\right)$	20.5857	17.0561	13.560	15.670	8.025	14.6313	20.2597	20.3431	18.3482
$2010\;\left(X_{3}\right)$	21.6142	17.4023	12.260	15.185	7.220	14.4179	19.6660	19.9031	16.2346
$2011\;\left(X_{4}\right)$	18.3904	15.2864	11.460	13.955	6.700	13.2390	18.1400	18.2282	15.2142
$2012\;\left(X_{5}\right)$	18.1590	15.1994	12.710	14.170	7.580	13.2382	18.2522	18.2864	17.4007
$2013\;\left(X_{6}\right)$	18.5324	15.6567	13.600	15.070	8.145	13.9860	18.8507	18.8497	18.5486
$2014\;\left(X_{7}\right)$	18.6472	15.7440	13.445	14.855	8.185	13.9803	19.0047	19.0099	18.2571
$2015\;\left(X_{8}\right)$	20.0291	16.4902	14.990	15.600	9.805	14.4142	19.5946	19.5970	21.1724
$2016\;\left(X_{9}\right)$	21.0013	17.4754	14.190	16.105	8.350	15.1890	20.8681	20.9305	19.6236
$2017\;\left(X_{10}\right)$	17.9236	14.8651	11.445	13.500	6.720	12.8552	17.5676	17.6466	15.2871
$2018\;\left(X_{11}\right)$	20.4016	16.9178	12.990	15.570	7.690	14.5733	20.0564	20.1505	18.0931
$2019\;\left(X_{12}\right)$	20.7732	17.4184	15.750	16.570	10.695	15.4182	21.0559	21.0423	21.9559
All years	19.7131	16.3365	13.210	15.095	7.970	14.1425	19.4223	19.4884	17.9655

**Table 5:** The 95% confidence intervals $\left(CI_{0.95}\right)$: $X_{1}\;(2008),\;\ldots ,\;X_{12}\;(2019)$.

Variable (Year)	Pairs of Estimators	$\mathrm{Lower}\;\mathrm{Limit}\;\mathrm{of}\;\mathrm{the}\;CI_{0.95}$ $\left(\mu g/m^{3}\right)$	$\mathrm{Upper}\;\mathrm{Limit}\;\mathrm{of}\;\mathrm{the}\;CI_{0.95}$ $\left(\mu g/m^{3}\right)$	$\mathrm{Length}\;\mathrm{of}\;\mathrm{the}\;CI_{0.95}$ $\left(\mu g/m^{3}\right)$
$X_{1}\;\left(2008\right)$	$\left(T\left(0.2\right),s^{W}\left(0.2\right)\right)$	19.8084	20.7331	0.9247
	$\left(T_{bi}\left(9\right),s_{bi}\left(9\right)\right)$	16.4104	17.1780	0.7676
$X_{2}\;\left(2009\right)$	$\left(T\left(0.2\right),s^{W}\left(0.2\right)\right)$	22.7226	23.7676	1.0450
	$\left(T_{bi}\left(9\right),s_{bi}\left(9\right)\right)$	19.9870	20.8588	0.8718
$X_{3}\;\left(2010\right)$	$\left(T\left(0.2\right),s^{W}\left(0.2\right)\right)$	22.6131	23.6382	1.0251
	$\left(T_{bi}\left(9\right),s_{bi}\left(9\right)\right)$	18.5928	19.4419	0.8491
$X_{4}\;\left(2011\right)$	$\left(T\left(0.2\right),s^{W}\left(0.2\right)\right)$	19.4984	20.4428	0.9444
	$\left(T_{bi}\left(9\right),s_{bi}\left(9\right)\right)$	17.1457	17.9259	0.7801
$X_{5}\;\left(2012\right)$	$\left(T\left(0.2\right),s^{W}\left(0.2\right)\right)$	21.2993	22.2385	0.9392
	$\left(T_{bi}\left(9\right),s_{bi}\left(9\right)\right)$	18.5019	19.2803	0.7784
$X_{6}\;\left(2013\right)$	$\left(T\left(0.2\right),s^{W}\left(0.2\right)\right)$	21.9628	22.9674	1.0046
	$\left(T_{bi}\left(9\right),s_{bi}\left(9\right)\right)$	19.5530	20.3653	0.8123
$X_{7}\;\left(2014\right)$	$\left(T\left(0.2\right),s^{W}\left(0.2\right)\right)$	23.2136	24.2042	0.9906
	$\left(T_{bi}\left(9\right),s_{bi}\left(9\right)\right)$	19.8670	20.6752	0.8081
$X_{8}\;\left(2015\right)$	$\left(T\left(0.2\right),s^{W}\left(0.2\right)\right)$	24.6840	25.7051	1.0211
	$\left(T_{bi}\left(9\right),s_{bi}\left(9\right)\right)$	22.1498	22.9828	0.8329
$X_{9}\;\left(2016\right)$	$\left(T\left(0.2\right),s^{W}\left(0.2\right)\right)$	22.7956	23.8797	1.0840
	$\left(T_{bi}\left(9\right),s_{bi}\left(9\right)\right)$	20.6076	21.5038	0.8962
$X_{10}\;\left(2017\right)$	$\left(T\left(0.2\right),s^{W}\left(0.2\right)\right)$	20.1329	21.0469	0.9140
	$\left(T_{bi}\left(9\right),s_{bi}\left(9\right)\right)$	17.4621	18.2149	0.7527
$X_{11}\;\left(2018\right)$	$\left(T\left(0.2\right),s^{W}\left(0.2\right)\right)$	22.6658	23.7046	1.0388
	$\left(T_{bi}\left(9\right),s_{bi}\left(9\right)\right)$	19.3850	20.2467	0.8618
$X_{12}\;\left(2019\right)$	$\left(T\left(0.2\right),s^{W}\left(0.2\right)\right)$	27.2138	28.3123	1.0985
	$\left(T_{bi}\left(9\right),s_{bi}\left(9\right)\right)$	24.3223	25.2218	0.8995

**Table 6:** Coefficient of skewness of $h_{i}$, i=1,…,12.

**Variable**	$h_{1}$	$h_{2}$	$h_{3}$	$h_{4}$	$h_{5}$	$h_{6}$
**Skewness**	0.2968	0.4070	0.2679	0.5104	0.4351	0.3874
**Variable**	$h_{7}$	$h_{8}$	$h_{9}$	$h_{10}$	$h_{11}$	$h_{12}$
**Skewness**	0.3426	0.4981	0.6277	−0.0688	0.1212	0.2379

**Table 7:** Statistical information on the transformed variables, $v_{i}$, i=1,…,12.

**Variable**	$v_{1}=\sqrt{h_{1}+40}$	$v_{2}=h_{2}$	$v_{3}=\sqrt{h_{3}+40}$	$v_{4}=\sqrt{h_{4}+20}$	$v_{5}=\sqrt{h_{5}+60}$	$v_{6}=\sqrt[{6.5}]{0.7\cdot h_{6}+80}$
**Mean**	8.8780	41.5695	9.0953	7.5570	9.6572	2.0441
**Variance**	0.7505	291.4980	0.8180	0.9817	0.4336	0.0005
***p*-Value**	0.3336	0.1765	0.8237	0.5638	0.1021	0.0161
**Skewness**	0.0093	0.4070	−0.0136	0.0799	0.1962	0.0071
**Variable**	$v_{7}=\sqrt[{6.5}]{0.5\cdot h_{7}+65}$	$v_{8}=\sqrt{h_{8}}$	$v_{9}=\sqrt[{7}]{h_{9}+50}$	$v_{10}={\left(h_{10}\right)}^{1.25}$	$v_{11}=h_{11}$	$v_{12}=h_{12}$
**Mean**	1.9789	7.0760	1.9548	146.9523	46.2482	43.3615
**Variance**	0.0003	0.6659	0.0018	3615.5855	345.2467	204.7272
***p*-Value**	0.0728	0.4168	0.0431	0.1592	0.6320	0.1607
**Skewness**	0.0027	0.0599	−0.2071	0.2300	0.1212	0.2379

**Table 8:** Coefficient of skewness of the variables *Working days*, *Saturday*, and *Sunday*.

**Variable**	*Working Days*	*Saturday*	*Sunday*
**Skewness**	0.4886	0.4596	0.6060

**Table 9:** Statistical information on the transformed variables, $u_{i}$, i=1,2,3.

**Variable**	$u_{1}=\sqrt[{6}]{0.5\cdot \left(Working\;days\right)+30}$	$u_{2}=\sqrt{Saturday+20}$	$u_{3}=\sqrt{Sunday+10}$
**Mean**	1.9225	8.0651	7.6110
**Variance**	0.0026	1.0587	1.1559
***p*-Value**	0.0524	0.2741	0.3501
**Skewness**	0.0469	0.0588	0.0976

## Data Availability

Data available in a publicly accessible repository.
